# Endoglin in head and neck neoplasms

**DOI:** 10.3389/fmed.2023.1115212

**Published:** 2023-02-10

**Authors:** Małgorzata Litwiniuk-Kosmala, Maria Makuszewska, Małgorzata Czesak

**Affiliations:** Department of Otorhinolaryngology, Head and Neck Surgery, Medical University of Warsaw, Warsaw, Poland

**Keywords:** endoglin, paraganglioma, angiogenesis, head and neck tumors, salivary gland tumors

## Abstract

Tumors of the head and neck region form a heterogeneous group of pathologies, including various benign lesions and malignant neoplasms. Endoglin, also known as CD105, is an accessory receptor for transforming growth factor beta (TGF-β), that regulates angiogenesis, both under physiological and pathological conditions. It is highly expressed in proliferating endothelial cells. Therefore, it is considered as a marker of tumor-related angiogenesis. In this review we discuss the role of endoglin as a possible marker of carcinogenesis, as well as a potential target for antibody-based therapies in the neoplasms of the head and neck region.

## Introduction

Tumors of the head and neck region form a heterogeneous group of pathologies, including benign lesions, such as hemangiomas, schwannomas or paragangliomas, and malignant neoplasms, among which squamous cell carcinoma occurs the most frequently. Since the head and neck compartment is the most vascularized anatomical region of the human body, a high level of vascularization is a common feature of the majority of tumors growing in this location. The location of the tumor in the head and neck region is very often surgically challenging, even in case of benign lesions. Therefore, apart from surgical resection, adjuvant treatment is often needed for tumor remnants or in case of recurrence. Hence, a lot of research has concentrated on characterizing the potential prognostic markers of those neoplasms and targets for molecular therapies. Endoglin, also known as CD105, has gained popularity as a marker of tumorigenesis, as well as a potential target for antibody-based therapy (1, 2). It is highly expressed in proliferating endothelial cells. Therefore, it is considered as a marker of tumor-related angiogenesis (3). In this review we summarize the role of endoglin in the pathophysiology of various types of benign, as well as malignant head and neck tumors. We also show the usefulness of endoglin as a prognostic marker and discuss the possibility of various diagnostic and therapeutic strategies based on anti-endoglin antibodies in the head and neck region.

## The role of endoglin in tumor angiogenesis: Possible diagnostic and therapeutic implications

Endoglin is a type I transmembrane glycoprotein that acts as an accessory receptor for transforming growth factor beta (TGF-β), a pleiotropic cytokine playing an important role in the regulation of cellular proliferation, differentiation, and migration. Human endoglin is a homodimeric protein with the molecular weight of 180 kDa. It contains a large extracellular domain (561 amino acids), a single transmembrane domain and a short cytosolic domain (4). Structurally, endoglin is a member of the ZP (zona pellucida) family of proteins that share a ZP domain in their extracellular region (5). The extracellular domain of endoglin also contains the tripeptide arginine-glycine-aspartic acid (RGD) motif, a sequence that serves as a biding site for many types of adhesive proteins (6). The ectodomain of endoglin may be released through proteolytic cleavage by matrix metalloproteinase 12 and 14 (MMP-12 and MMP-14) and is present in the circulation as a soluble form of endoglin (Sol-eng) (7). Small amounts of Sol-eng are also present in the serum of healthy subjects. However, an elevated level of Sol-eng was reported in the serum of patients with breast, liver and colorectal cancer, as well as non-small cell lung carcinoma (8–10).

**FIGURE 1:**
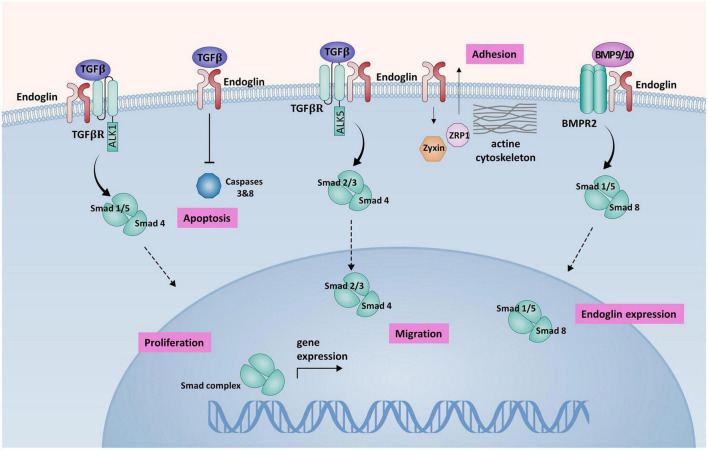
A schematic presentation of endoglin-mediated signaling in endothelial cells (ECs). Endoglin associates with TGF-βRII and ALK1 forming a receptor complex, that binds TGF-β1. Activation of this receptor complex leads to the phosphorylation of Smad1/5 that form a complex with co-Smad (Smad4). Smad complex is then translocated to the nucleus, where it regulates the expression of target genes, promoting cell proliferation. Association of endoglin with the ALK5/TGF-βII receptor complex inhibits Smad2/3 dependent signaling pathway, that has an antiproliferative effect on ECs. Endoglin promotes BMP9/10 signaling *via* BMPR2 receptor complex. Activation of BMPR2 receptor complex leads to Smad1/5/8 phosphorylation and subsequent increased endoglin expression in a positive feedback loop. Binding of TGF-β by endoglin induces inhibition of apoptosis *via* caspases 3 and 8 inhibition. Endoglin interaction with ZRP-1 and Zyxin regulates cell migration, inducing the reorganization of actin cytoskeleton and promoting cell adhesion (57).

Endoglin modulates the activity of TGF-β mostly *via* activin-like kinase 1 (ALK1) and activin-like kinase 5 (ALK5) receptors that belong to the superfamily of TGF-β type I receptors (TGF-βR1) (11). These type I receptors activate signaling pathways *via* Smad-1, –5, and –8 (ALK1) or Smad2 and –3 (ALK5), regulating the expression of various genes involved in angiogenesis (6). Studies showed that the balance between the ALK1 and ALK5 signaling pathways in endothelial cells (ECs) plays a crucial role in angiogenesis and vascular remodeling (12). The overexpression of endoglin in ECs counteracts the antiproliferative effect of TGF-β1 (13). Endoglin also has a protective role against apoptosis induced by hypoxia and TGF-β1 in ECs (13, 14). All these findings support the hypothesis that endoglin and ALK1 participate in a common signaling pathway that is crucial for EC response to TGF-β family members (Figure 1).

The main function of endoglin involves the regulation of angiogenesis, both under physiological and pathological conditions. It was demonstrated that the endoglin knockout mice died early in the embryonic development process due to various vascular defects (15). In addition, mutations in the endoglin gene were identified in patients with hereditary hemorrhagic telangiectasia (HHT), an autosomal dominant vascular dysplasia (16, 17). The expression of endoglin is particularly high in actively proliferating ECs. Studies showed, that anti-endoglin antibodies are more specific in staining proliferating ECs than other endothelial markers, such as CD31, CD34, or VEGFR (18). All these data suggest that endoglin might be an excellent marker of tumor vascularization. Anti-endoglin antibodies have been used for microvessel density (MVD) calculation in various types of tumors. It was demonstrated that MVD assessed on the basis of endoglin immunohistochemical expression in the tumor tissue correlated with poor prognosis in colorectal, breast, prostatic and lung cancer patients. Such a correlation was not observed when MVD was calculated using the anti-CD34 or anti-CD31 antibodies, which are traditional markers of angiogenesis (19, 20).

Tumor angiogenesis is an important target in cancer therapy. The first approved antiangiogenic drugs, such as bevacizumab, sorafenib and sunitinib, are anti-VEGF (vascular endothelial growth factor) agents. Nevertheless, the inhibition of non-VEGF angiogenic pathways is an interesting strategy that may address tumor resistance to anti-VEGF therapies. TRC105 (TRACON Pharmaceuticals Inc.) is a chimeric IgG1 anti-endoglin antibody, that induces apoptosis in endoglin-positive tumor cells. Its safety, tolerability and antitumor activity have already been demonstrated in several phase I clinical trials in patients with advanced, refractory solid tumors (21). According to a study where TRC105 was combined with bevacizumab, several patients who had previously progressed on anti-VEGF therapies experienced reductions in tumor volume or remained progression-free for a longer period than on bevacizumab alone. TRC105 in combination with bevacizumab is now tested in randomized phase II trials in glioblastoma and renal cell carcinoma patients (22, 23).

## Endoglin as a marker in various malignant and non-malignant head and neck tumors

### Head and neck squamous cell carcinoma

Head and neck squamous cell carcinoma (HNSCC) includes cancers of the oral cavity, nasopharynx, oropharynx, hypopharynx and larynx. Over the years, significant progress has been made in the diagnosis and treatment of HNSCC, yet the 5-year overall survival rate remains unsatisfactory (24). The basic prognostic factors include the histopathological type of the tumor, its location, size, depth of infiltration, presence of lymph node metastases, tobacco use and human papillomavirus (HPV) infection in case of oropharyngeal cancer, or Epstein-Barr (EBV) virus in nasopharyngeal carcinoma (25). In addition to the described prognostic factors, immunohistochemical markers such as p53, Ki-67, p16, cyclin D1, and vessel density (MVD) calculation play a role in the prognostic evaluation and therapeutic decisions (26). Increased MVD values, recognized as an independent prognostic indicator, reflect the progression of the disease and a shorter disease-free survival rate (DSF). In case of HNSCC, some studies confirmed this correlation with MVD, while others did not find such a correlation. The differences result from the use of different protocols in immunohistochemical staining for endothelial marker studies. Endothelial targets, such as CD31, CD34 and, increasingly, endoglin, which is also involved in angiogenesis, were used as endothelial targets for MDV calculation in HNSCC (27, 28).

Schimming and Marme (29) examined endoglin in the tumor tissue of oral cancer at different TNM stages and in the normal mucosa. TNM is an international classification used for cancer staging. T1-4 describes the tumor size and its local spread to surrounding tissues, N0-3 describes lymph node metastases, whereas N + means any lymph nodes positive for metastases. M0-1 stands for distant metastases. In the reported study endoglin expression was significantly higher in the neoplastic tissue, and it was lower in T1 compared to other T stages, as well as in N0 compared to N + cases. Moreover, higher endoglin expression was observed in moderately differentiated compared to poorly differentiated tumors (29). Nagatsuka et al. (30) investigated endoglin, as well as CD31 and CD34 antibodies in the normal oral mucosa and in 40 cases of HNSCC to study the properties and morphology of blood vessels. Endoglin-positive endothelial cells were found in the neo-vessels of the oral cavity squamous cell carcinomas (SCC) with extensive remodeling and in immature SCC neo-vessels (30).

Chuang et al. (31) demonstrated a high expression of endoglin in biopsy tissues collected from 94 patients with advanced oral SCC and N + cases. In addition, cumulative 5-year disease-free survival (DFS) correlated significantly with low endoglin and VEGF expression (31). Similar results were obtained in other studies on endoglin expression in oral SCC (32, 33).

Endoglin and VEGF testing was performed by Chen et al. (34) in the postoperative specimens of SCC of the hypopharynx. The study showed a high expression of endoglin in N + cases and in clinically advanced tumors. It was reported that the overall 5-year survival rate for patients with low endoglin expression was higher than in patients with high endoglin expression, and high endoglin expression was found to be an independent survival factor (34).

Marioni et al. (35) analyzed MDV by endoglin immunohistochemistry for laryngeal SCC in forty-three patients undergoing partial or total laryngectomy. The study showed that disease recurrence was correlated with endoglin-assessed MVD. Furthermore, it was confirmed that endoglin-assessed MVD might be used as a predictive parameter for patients at an increased risk of local and regional recurrence of laryngeal SCC (35, 36). Zvrko et al. (37) investigated the expression of endoglin in 40 cases of postoperative supraglottic part of larynx carcinoma specimens. The study showed statistical correlation between high endoglin-assessed MVD and advanced clinical stage, pN + and loco-regional recurrence. Moreover, endoglin-assessed MVD was found to be the only independent predictor of recurrence (37). The study was also performed on 40 glottal SCC biopsies. A significant correlation was confirmed between endoglin-assessed MVD and advanced pT stage and clinical stage as well as recurrence. Also, high endoglin-assessed MVD was associated with poorer DFS (38). In Marioni et al. (27) analyzed MVD *via* endoglin and CD31 in forty-five paired SCC biopsies and surgical specimens of the larynx. The median endoglin-assessed MVD was shown to be higher in N + cases. Statistical analysis revealed that DFS correlated with endoglin and CD31-assessed MVD in both biopsies and surgical specimens. The multivariate Cox regression showed that the pathological grade and endoglin-assessed MVD predicted DFS in SCC of the larynx. The authors emphasized that further research was required to determine the role of endoglin-assessed MVD as a prognostic marker suitable for identifying patients at a higher risk of recurrence requiring more aggressive treatment and clinically N0 patients requiring elective neck dissection (27).

### Paragangliomas

Paragangliomas are rare, usually benign tumors, derived from the autonomic nervous system paraganglia. Paragangliomas of the head and neck region most commonly develop in the carotid body. Less common anatomical locations include the middle ear (tympanic paragangliomas), jugular fossa (jugular paragangliomas) and vagus nerve (vagus paragangliomas). Since head and neck paragangliomas arise from parasympathetic ganglia, they very rarely secrete catecholamines, unlike adrenal paragangliomas (pheochromocytomas), that are derived from the sympathetic system (39). Approximately 30% of paragangliomas have a familial occurrence, so wide genetic studies were conducted to discover the genetic background of the tumors (40). Germline and somatic mutations that lead to paraganglioma development may be classified into three clusters: pseudohypoxia-related genes (clusters 1A and 1B), kinase signaling–related genes (cluster 2) and Wnt signaling–related genes (cluster 3). Cluster 1 is divided into A and B subcategories according to the position of the mutation either in the Krebs cycle or hypoxia signaling pathway (41). The most common mutations in the cluster 1 are located in genes coding the succinate dehydrogenase enzyme complex (SDH), which participates in the citric acid cycle and mitochondrial electron transport chain. Mutations in the *SDHx* genes trigger a state of pseudohypoxia, which leads to the activation of the hypoxia-inducible factor 1-a (HIF-1α) transcription factor pathway. The activation of the HIF-1α signaling pathway leads to the upregulation of endoglin expression (42). Eleno et al. (43) were the first to assess the level of endoglin expression in cervical paragangliomas. They reported a significantly higher expression of endoglin in paraganglioma tissues than in the control lung tissue, whereas VEGF level was similar in both tissues. Endoglin was almost exclusively expressed on endothelial cell surface, without any staining of tumor parenchymal cells. Recently, in our center, we have also investigated the expression of endoglin in various types of head and neck paragangliomas, as well as the level of Sol-eng in the patients’ serum (44). The results of this study showed a high level of endoglin expression in tumor samples. The level of Sol-eng in serum samples was significantly higher in the tumor group than in healthy controls and a positive correlation with the tumor size was observed. In the examined group of patients, a complete surgical resection led to the reduction of Sol-eng level to the values obtained in control group 4 weeks after the operation.

### Salivary gland tumors

Malignant salivary gland tumors are characterized by a highly heterogeneous histological structure, with over 20 histopathological types included in the WHO classification. Unpredictable clinical behavior is their unique clinical feature, with distant metastases that may occur even many years after the initial diagnosis and treatment. Several authors assessed angiogenesis in salivary gland tumors, using endoglin as a marker, in order to improve knowledge about the prognosis and adequate management (45–48). Endoglin-positive vessels were absent in the normal salivary gland tissue and rare in pleomorphic adenomas. However, Warthin’s tumors, that are the most frequent benign tumors of parotid gland, presented very high endoglin expression in their lymphoid component (47). A significant increase in endoglin expression was observed in malignant tumors. It was the highest in mucoepidermoid carcinomas, where it reached 83–85% (45, 48) and less common in polymorphous low-grade adenocarcinomas, where 42% increase of endoglin expression was noted (45). The results for adenoid cystic carcinoma vary from 65% in a study by Tadbir et al. (48) to 8% in a study by Cardoso et al. (45). Such differences between studies may result from dissimilar microscopic tumor subtypes. However, no significant difference was found in endoglin expression comparing adenoid cystic carcinoma with and without high-grade transformation (49). Differences in endoglin expression in various types of malignant salivary gland tumors may be explained by their variable myoepithelial differentiation, although no correlation was demonstrated between the degree of angiogenesis and the amount of myoepithelial cells (50). Carcinomas with myoepithelial differentiation, regardless of the amount of myoepithelial cells, were associated with a significantly lower vascular density (51). The highest density of endoglin-positive blood vessels was also observed in tumor stromal areas with marked inflammation (46).

Fonseca et al. (47) did not reveal a significant correlation between endoglin expression and clinicopathological parameters. Moreover, vascular density did not correlate with the survival rates of patients affected by malignant salivary gland tumors (47). The expression of endoglin was also very similar between non-metastasizing and metastasizing primary malignant salivary gland tumors in the study by Cardoso et al. (45) but adenoid cystic carcinomas with endoglin-positive vessels were characterized by an increased risk for metastasis. Gleber-Netto et al. (51) conducted a study concerning minor salivary gland mucoepidermoid carcinoma. They assessed endoglin expression and showed a greater angiogenic activity measured in intratumoral than in peritumoral areas. Contrary to observations in other neoplasms, recurrence and nodal metastasis were associated with low neo formed vessel density, indicating that impaired angiogenesis could lead to an aggressive phenotype (51).

### Vascular tumors

Endoglin is also highly expressed in the head and neck tumors of vascular origin, such as capillary hemangiomas and juvenile nasopharyngeal angiofibroma (52, 53). In a study by Matsumoto et al. (52) all capillary hemangioma cases showed moderate-to-strong endoglin staining of blood vessel endothelial cells. The staining score was significantly higher than in normal controls (normal oral mucosa) and cavernous hemangiomas (52). The endothelial cells of capillary hemangiomas have an active proliferative capacity, reflected by significantly elevated Ki-67 expression. Furthermore, the expression of VEGF-a and COX-2 in the stromal fibroblasts and macrophages was stronger in capillary hemangiomas than in the control tissue or cavernous hemangiomas. Wang et al. (53) performed endoglin-based MVD (endoglin/MVD) measurements in juvenile angiofibroma patients. The analysis of the results with clinicopathological features showed a correlation between endoglin/MVD and angiofibroma recurrence. endoglin/MVD was a better predictor of disease recurrence after curative resection than other clinicopathological features (53).

### Rhabdomyosarcoma

Endoglin expression is also considered to be promising prognostic marker in rhabdomyosarcoma (54, 55). Radzikowska et al. (54) analyzed microvessel density based on CD31, CD34, and endoglin expression in 49 cases of pediatric rhabdomyosarcoma (RMS). CD31, CD34, and endoglin were expressed in all RMS cases (54). Endoglin/MVD was significantly higher in patients with alveolar RMS and those with metastatic disease. Patients with higher levels of endoglin/MVD were at a higher risk of death. The authors concluded that endoglin was a relevant angiogenesis marker in pediatric RMS, and endoglin/MVD was an independent risk factor of short overall survival in children with RMS. The expression of endoglin was also predictive of aggressive biologic behavior of non-melanoma skin cancers – basal and squamous cell carcinomas located mainly in the head and neck region (55). Most of the examined tumors exhibited negative-to-weak endoglin staining but a statistically significant correlation was found between tumor local recurrence and endoglin expression.

### Vestibular schwannomas

Endoglin expression was also studied in a series of NF2-associated vestibular schwannomas (VSs), as compared to a group of sporadic VSs (56). No significant differences were found between NF2-associated VSs and sporadic cases in terms of endoglin expression. A positive correlation was observed between tumor growth rate (measured on contrast-enhanced MRI) and vessel density based on endoglin staining, but only in NF2-associated VSs.

## Conclusion

An increasing number of studies point to endoglin as a potential marker in various types of head and neck neoplasms (Table 1). It was shown that MVD calculated using endoglin staining was a reliable marker of more advanced disease, lymph node metastases and poor prognosis in HNSCC and in rhabdomyosarcoma. In addition, several studies demonstrated that a high expression of endoglin in tumor tissues was an independent risk factor of lower 5-year overall survival rate (Table 1). Endoglin is also highly expressed in non-malignant richly vascularized tumors, including paragangliomas and juvenile angiofibromas. All the data considered collectively make endoglin a promising target for biological antibody-based therapy, especially in recurrent and advanced cases. However, even though TRC105, the anti-endoglin antibody was tested as a potential therapeutic agent in various solid tumors, there is a lack of similar studies in head and neck neoplasms. Future research directions in this area should also include studies on a soluble form of endoglin as a potential marker of tumor progression and recurrence.

**TABLE 1:** The expression of endoglin in various benign and malignant head and neck tumors, reported in different studies.

Benign head and neck tumors			
**Type of tumor**	**Outcome**	**Clinical implication**	**References**
Paraganglioma	High expression		(43, 44)
Warthin’s salivary gland tumor	High expression		(45)
Pleomorphic adenoma	Low expression		(47)
Capillary hemangioma	High expression		(52)
Juvenile angiofibroma	High expression	Positive correlation with tumor recurrence	(53)
Schwannoma	High expression	Positive correlation with tumor growth rate	(56)
**Malignant head and neck neoplasms**			
Oral cancer	High expression		(29)
Oral cancer	High expression		(30)
Oral cancer	High expression	5-year DFS correlated with high endoglin expression	(31)
Hypopharyngeal cancer	High expression in N+ and advanced cases	Independent survival factor	(32)
Laryngeal cancer	High expression	Marker of recurrence	(35)
Laryngeal cancer	High expression in N+ and advanced cases	Marker of recurrence	(36)
Laryngeal cancer	High expression in N+ and advanced cases	5-year DFS predictor	(37, 38)

The expression of endoglin was measured in tumor tissue. If the level of endoglin expression was correlated with clinical data, the results of this correlation was included in “clinical implication” column. N+, cases with lymph node involvement; DFS, disease free survival.

## Author contributions

ML-K contributed to the conception and design of the manuscript and prepared the table and figure. ML-K, MM, and MC wrote the sections of the manuscript. All authors contributed to the manuscript revision, read, and approved the submitted version.
